# Heterogeneous in vitro effects of doxorubicin on gene expression in primary human liposarcoma cultures

**DOI:** 10.1186/1471-2407-8-313

**Published:** 2008-10-29

**Authors:** Daigeler Adrien, Klein-Hitpass Ludger, Chromik Ansgar Michael, Müller Oliver, Hauser Jörg, Homann Heinz-Herbert, Steinau Hans-Ulrich, Lehnhardt Marcus

**Affiliations:** 1 Department of Plastic Surgery, Burn Center, Hand surgery, Sarcoma Reference Center, BG-University Hospital Bergmannsheil, Ruhr University Bochum, Bürkle-de-la-Camp-Platz 1, 44789 Bochum, Germany; 2 Institute of Cell Biology (Tumor Research), IFZ, University of Essen, Virchowstr. 173, 45122 Essen, Germany; 3 Department of Surgery, St. Josef Hospital, Ruhr-University, Bochum, Germany; 4 Tumor Genetics Group, Max-Planck-Institut für molekulare Physiologie, Otto Hahnstr. 11, 44227 Dortmund, Germany

## Abstract

**Background:**

Doxorubicin is considered one of the most potent established chemotherapeutics in the treatment of liposarcoma; however, the response rates usually below 30%, are still disappointing. This study was performed to identify gene expression changes in liposarcoma after doxorubicin treatment.

**Methods:**

Cells of 19 primary human liposarcoma were harvested intraoperatively and brought into cell culture. Cells were incubated with doxorubicin for 24 h, RNA was isolated and differential gene expression was analysed by the microarray technique.

**Results:**

A variety of genes involved in apoptosis were up and down regulated in different samples revealing a heterogeneous expression pattern of the 19 primary tumor cell cultures in response to doxorubicin treatment. However, more than 50% of the samples showed up-regulation of pro-apoptotic genes such as *TRAIL Receptor2*, *CDKN1A*, *GADD45A*, *FAS*, *CD40*, *PAWR*, *NFKBIA*, *IER3*, *PSEN1*, *RIPK2*, and *CD44*. The anti-apoptotic genes *TNFAIP3*, *PEA15*, *Bcl2A1*, *NGFB*, and *BIRC3 *were also up-regulated. The pro-apoptotic *CD14*, *TIA1*, and *ITGB2 *were down-regulated in more than 50% of the tumor cultures after treatment with doxorubicin, as was the antiapoptotic *YWHAH*.

**Conclusion:**

Despite a correlation of the number of differentially regulated genes to the tumor grading and to a lesser extent histological subtype, the expression patterns varied strongly; however, especially among high grade tumors the responses of selected apoptosis genes were similar. The predescribed low clinical response rates of low grade liposarcoma to doxorubicin correspond to our results with only little changes on gene expression level and also divergent findings concerning the up- and down-regulation of single genes in the different sarcoma samples.

## Background

Together with malignant fibrous histiocytoma (not otherwise specified sarcoma, NOS), liposarcoma represents the most common entity of soft tissue sarcomas and accounts for approximately 20% of sarcomas in adults [1-3]. Although surgery and radiation therapy could achieve good results concerning local control, distant metastatic disease remains a therapeutic dilemma limiting survival [4,5]. With a maximum response rate of approximately 20% the effects of cytostatics on liposarcoma are still disappointing [6-8]. The most favoured chemotherapeutics for treatment of advanced soft tissue sarcoma, including liposarcoma, are ifosfamide and doxorubicin, but the data for ifosfamide differ with respect to improvement of local control and survival [9-11]. Although meta-analysis of 14 randomised trials found that doxorubicin treatment was associated with a 10% improvement of recurrence free survival, the overall survival could not be improved [12-14]. In carcinomas, multiple mechanisms of drug resistance on the molecular level have been characterized [15,16] including over-expression of *p53 *[17-20], *MDR1 *(multidrug resistance gene 1) [20-22], *MRP1 *(multidrug resistance-associated protein), the induction of DNA repair [20] and many others involving tumor suppressor genes, oncogenes, cell cycle regulators, transcription factors, growth factor receptors, and cell death regulators. Only little is known about the molecular basis of drug resistance in soft tissue sarcomas and studies on the effect of cytostatics on gene expression, especially in liposarcomas [23-27], are rare. Comprehensive knowledge of the differential expression patterns induced by cytotoxic drugs may be useful for examining the molecular basis of drug effects and also drug resistance. Because of the limited comparability of established purchasable sarcoma cell lines to in vivo tumors, we primarily harvested liposarcoma cells from resection specimens, incubated the cultured cells with doxorubicin and evaluated the changes in gene expression with a focus on genes related to apoptotic pathways. To the authors' knowledge, to date there are no studies that examined the effects of doxorubicin on primary human liposarcoma on a molecular basis.

## Methods

Primary human liposarcoma tumor samples of at least 1 cm^3 ^were harvested intraoperatively from patients undergoing resection of an already diagnosed liposarcoma and immediately processed under sterile conditions. Seven atypical lipomas (low grade sarcomas), four dedifferentiated, four pleomorphic, three myxoid/round cell, and one myxoid liposarcoma were included. The grading of the tumors ranged from GI to GIII (GI: 4, GII: 8, GIII: 7). The probes were derived from primary tumors in 12, from recurrent tumors in six, and from metastasis in one case. Nineteen primary human liposarcoma cultures were isolated by dissecting the tumor and digesting the minced samples enzymatically with 10 ml each of collagenase and dispase (10 mg/ml). The single cell suspension was depleted of red blood cells and cellular debris by centrifugation through a Ficoll-Hypaque density gradient. Liposarcoma cells were diluted and cultured during the whole experiment with Leibovitz's L-15 medium, supplemented with 2.0 mM glutamine and 10% fetal bovine serum in a humidified atmosphere in free air exchange with atmospheric air. Cells were seeded at a density of 2 × 10^6 ^in 25 cm^2^flasks; 24 h later, after having grown to a subconfluent layer, cell cultures were incubated with doxorubicin (0.5 μg/ml) for 24 h and equal volume of PBS as control [28-30].

### Oligonucleotide microarray analysis

For microarray analyses we used the Affymetrix Gene Chip platform employing a standard protocol for sample preparation and microarray hybridization that has been described in detail previously [26,31]. Briefly, total RNA was converted into double-stranded cDNA using an oligo-deoxythymidine primer containing the T7 RNA polymerase binding site (5'-GCATTAGCGGCCGCGAAATTAATACGACTCACTATAGGGAGA – (dT)_21_V-3') (MWG Biotech, Ebersberg, Germany) for first strand synthesis. After generation of double-stranded cDNA from the first-strand cDNA, biotinylated cRNA was synthesized by *in vitro *transcription using the BioArray High Yield RNA Transcript Labeling Kit (Enzo Diagnostics, New York, USA). Labeled cRNA was purified on RNeasy columns (Qiagen, Hilden, Germany) and fragmented and hybridized to HG-U133A microarrays (Affymetrix, Santa Clara, USA). The arrays were washed and stained according to the manufacturer's recommendation and finally scanned in a GeneArray scanner 2500 (Agilent, Santa Clara, USA).

Array images were processed to determine signals and detection calls (Present, Absent, Marginal) for each probeset using the Affymetrix Microarray Suite 5.0 software (MAS 5.0; statistical algorithm). The clustering was performed unsupervised. Pairwise comparisons of treated *versus *control samples were carried out with MAS 5.0, which calculates the significance (change p-value) of each change in gene expression based on a Wilcoxon ranking test. To limit the number of false positives, we restricted further target identification to those probesets, which received at least one present detection call in the treated/control pair. Each single microarray analysis was derived from one cell culture. The treated cells were compared to the control. Probesets exhibiting a signal log2 ratio > 1.0 and a change p-value < 0.004 or a signal log2 ratio < -1.0 and a change p-value > 0.996 (corresponding to 2-fold up- or down-regulation) were identified by filtering using the Affymetrix Data Mining Tool 3.0 (Table 1). Additionally unsupervised clustering was performed between the 19 control tumor samples.

**Table 1:** Summarized patients' data

Patient	Gender	Age at operation	Site	Size in cm	Histological subtype	Responder type	Grading	Specimen character	Previous radiation	Previous chemotherapy
1	female	69 years	lower arm	4.5 × 3.5 × 2.2	atypical lipoma with partly dedifferentiated areas	low	G2	local recurrence	no	no
2	female	74 years	Thigh	14.5 × 7.5 × 9	myxoid/roundcell liposarcoma	high	G3	primary tumor	no	no
3	female	70 years	upper arm	3 × 5 × 6	atypical lipoma	low	G1	primary tumor	no	no
4	male	74 years	Thigh	16.5 × 9 × 7	dedifferentiated liposarcoma	medium	G2	primary tumor	no	no
5	male	38 years	Knee	8.3 × 4 × 7	myxoid liposarcoma	high	G3	primary tumor	yes	no
6	female	58 years	pelvis retro-peritoneal	4 × 7 × 9	myxoid/roundcell liposarcoma	high	G3	metastasis	no	no
7	male	37 years	Thigh	7 × 14 × 9	myxoid/roundcell liposarcoma	medium	G2	primary tumor	no	yes
8	female	85 years	lower arm	11 × 8 × 4	pleomorphic liposarcoma	high	G3	primary tumor	no	no
9	male	53 years	Thigh	10 × 3 × 5	atypical liposarcoma	low	G2	local recurrence	yes	no
10	male	76 years	Thigh	3.5 × 3 × 3	dedifferentiated liposarcoma	high	G3	local recurrence	yes	no
11	female	57 years	Thorax	4.9 × 4 × 3	pleomorphic liposarcoma	high	G3	local recurrence	no	no
12	female	76 years	Thigh	38.5 × 17.5 × 6	atypical lipoma	low	G1	primary tumor	no	no
13	female	74 years	Thigh	7 × 6 × 4	dedifferentiated liposarcoma	medium	G2	primary tumor	no	no
14	female	70 years	Thorax	1.9 × 1.3 × 1	pleomorphic liposarcoma	medium	G2	residual tumor	no	no
15	male	70 years	Thigh	9 × 3 × 6	atypical lipoma	medium	G1	primary tumor	no	no
16	male	60 years	Thigh	7.5 × 6 × 5.5	pleomorphic liposarcoma	high	G3	primary tumor	no	no
17	female	78 years	Thigh	13 × 10 × 6	atypical lipoma with partly dedifferentiated areas	high	G2	local recurrence	yes	no
18	female	67 years	Thigh	35 × 15 × 12	atypical lipoma	low	G1	primary tumor	no	no
19	male	60 years	upper arm	9,9 × 7 × 7	dedifferentiated liposarcoma	medium	G2	local recurrence	yes	no

Genes associated with apoptotic pathways were selected based on Gene Ontology (GO)-analysis [32].

Expression changes were correlated to the grading and the histological sub-entity of the tumors. Only tumor samples were included in the final analysis whose gross sections were diagnosed as liposarcomas by an experienced soft tissue pathologist.

According to the number of differentially expressed genes after incubation with doxorubicin, liposarcomas were categorized into high (n > 2000), intermediate (100 < n < 1000) and low responders (n < 100). The results were uploaded to NCBI GEO, number GSE12972 .

### Real-time PCR for microarray data validation

Microarray data validation was performed for selected gene products with relevant up-regulation in more than ten out of the 19 liposarcoma probes (CD40, CDKN1A, FAS, GADD45A, NFKBIA, PAWR, TNFAIP3, and TNFRSF10B) or relevant down-regulation in at least ten probes (YWHaH, PPP3CA, and ITGB2). CD14 and TIA were not tested because no high quality PCR assays were purchasable.

Total RNA (2 μg) was reverse transcribed using the High Capacity cDNA Archive Kit (Applied Biosystems). Realtime PCR was done with a 7900HT SDS system (Applied Biosystems) in 20 μl reaction volume containing 1× Master Mix, 1 μl assay and cDNA equivalent to 2 ng total RNA. All reagents and realtime PCR assays (m1 type only; CD40_Hs00386848_1, CDKN1A_Hs00355782_m1, FAS_Hs00236330_m1, GADD45A_Hs00169255_m1, ITGB2_Hs00164957_m1, NFKBIA_Hs00153283_m1, PAWR_Hs00169332_m1, PPP3CA_Hs00174223_m1, TNFAIP3_Hs00234713_m1, TNFRSF10B_Hs00366272_m1, YWHaH_Hs00607046_m1) used were purchased from Applied Biosystems. Reactions were performed in duplicates and analysed by the deltadeltaCT method. Human GAPD was used for normalization. Pearson correlation of the microarray and the rtPCR data was calculated using SPSS Version 15.0 for Windows (SPSS Inc., Chicago, USA).

## Results

Sixteen tumors were located at the extremities, three at the trunk. The tumors diameters ranged from 1 cm to 38,5 cm. Twelve primary cultures were harvested from primary tumors, 6 from local recurrences, and 1 from a metastatic tumor. The majority of tumors were high grade (G2-3); only 4 were diagnosed low grade (G1). Atypical lipoma or highly differentiated liposarcoma was the most common histological subentity (n = 7) followed by dedifferentiated (n = 4) and pleomorphic (n = 4), myxoid/rundcell (n = 3) and myxoid (n = 1) liposarcoma. All low grade tumors were diagnosed as atypical lipoma, whereas 3 atypical lipomas were categorized as G2 tumors because of aggressive growth or localized areas of dedifferentiation. The other subentities were all categorized as high grade tumors (G2-3). A summary of the patients' data is given in table 1.

**Figure 1:**
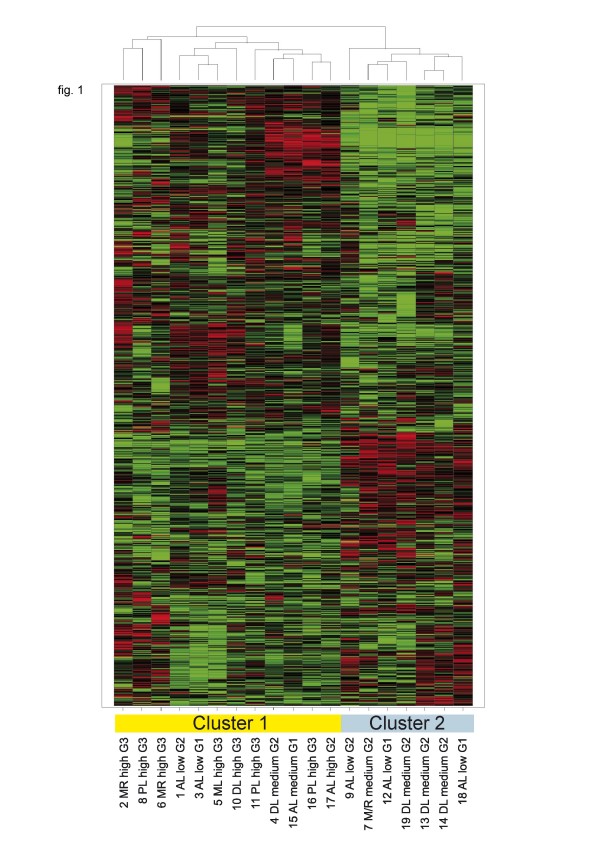
**Unsupervised hierarchical cluster analysis of the 19 primary tumor cell cultures without doxorubin treatment. **Gene signal intensities were normalized to the mean signal of all samples, log2 transformed and subjected to hierarchical clustering (UPGMA, Spotfire) and correlation as a similarity measure. Horizontal rows represent individual genes; vertical columns represent individual samples. Black indicates average signal intensity, brightest red ≥ 4-fold up-regualtion, brightest green ≥ 4-fold down-regulated gene expression relative to the mean. Only probesets receiving P detection calls in 6 or more samples and a stander deviation of normalized signals > 0.2 were considered. (7239 retained). The dendogram at the top of the matrix indicates the degree of similarity between tumor samples (the higher the dendogramm, the lower the similarity). Two major clusters were identified as indicated. MR: Myxoid/Roundcell Liposarcoma, PL: Pleomorphic Liposarcoma, AL: Atypical Lipoma, ML: Myxoid Liposarcoma, DL: Dedifferentiated Liposarcoma, low: low responder group, medium: medium responder group, high: high responder group.

Hierarchical clustering of expression profiles from untreated samples did not clearly separate according to histological subtype or grading, but revealed two major branches, which showed some enrichment for G3 (left) and G2 tumors (right) (fig. 1).

**Figure 2:**
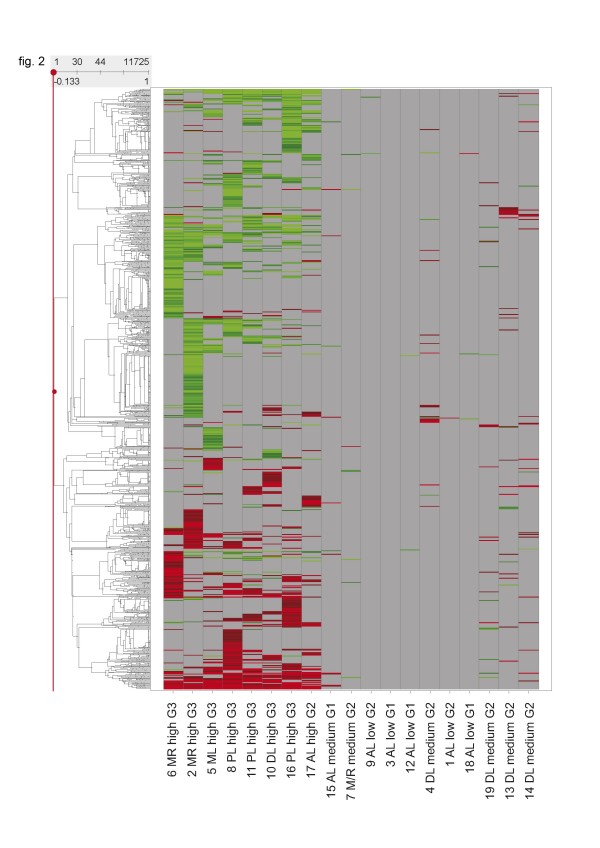
**Heatmap of total gene expression changes and cluster analysis after incubation with doxorubicin for 24 h**. Horizontal rows represent individual genes; vertical columns represent individual samples. Color range: Brightest red (Change call increased (change p-value < 0.002) and Signal Log Ratio > 1): SLR = >2 (4× or higher), Black: SLR = 0 (no change); not visible as a consequence of the filtering process, Brightest green (Change call decreased (change p-value > 0.998) and Signal Log Ratio < -1): SLR < -2 (0.25× or smaller). Grey: no value (requirements for a reliably measured target not met). Calculated in Affymetrix comparison analysis (MAS5.0 algorithm) and at least one present call in the two síngle array analyses compared in the comparison analysis. The dendogram at the left side indicates the degree of similarity among the selected genes according to their expression patterns (the higher the dendogramm, the lower the similarity). MR: Myxoid/Roundcell Liposarcoma, PL: Pleomorphic Liposarcoma, AL: Atypical Lipoma, ML: Myxoid Liposarcoma, DL: Dedifferentiated Liposarcoma, low: low responder group, medium: medium responder group, high: high responder group.

According to the number of differentially expressed genes n after incubation with doxorubicin, the 19 liposarcomas were categorized into high (n > 2000), intermediate (100 < n < 1000) and low responders (n < 100). All poorly differentiated (G3) tumors were high responders; G2 tumors were predominantly intermediate responders; and, most of the G1 tumors were low responders. A heatmap of all differentially expressed genes after 24 h of doxorubicin tratment shows heterogeneous response patterns (fig. 2).

Correlating the overall expression changes with the histological subtype showed that most of the atypical lipomas were low responders (5 low, 1 medium, 1 high), dedifferentiated were predominantly medium responders (3 medium, 1 high), most myxoid/roundcell and myxoid liposarcomas as well as the pleomorphic liposarcomas were high responders (3 high, 1 medium each). The high grade sarcomas (G3) clustered closely together.

**Figure 3:**
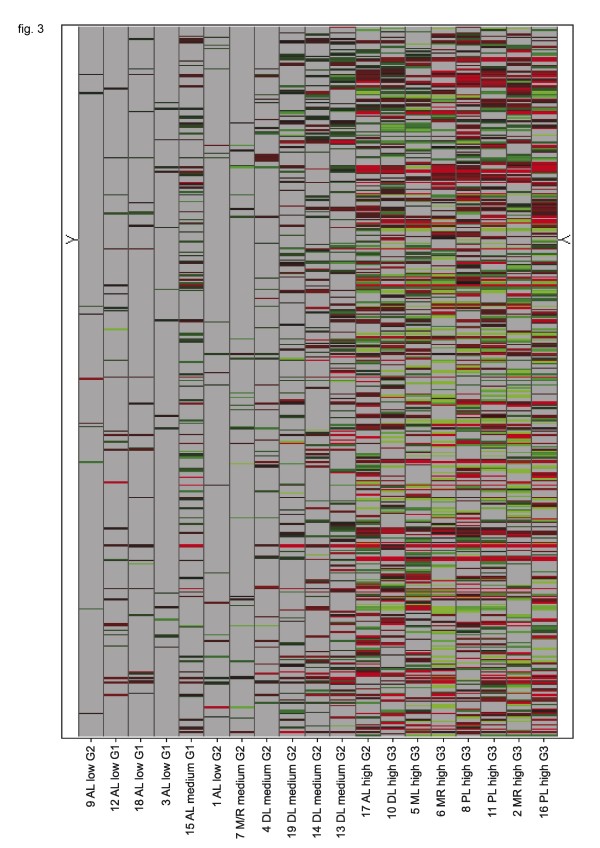
**Heatmap of expression changes of genes related to apoptotic pathways after incubation with doxorubicin for 24 h**. Horizontal rows represent individual genes; vertical columns represent individual samples (left to right: low responders to high responders). Color range: Brightest red (Change call increased (change p-value < 0.002) and Signal Log Ratio > 1): SLR = >2 (4× or higher), Black: SLR = 0 (no change); not visible as a consequence of the filtering process, Brightest green (Change call decreased (change p-value > 0.998) and Signal Log Ratio < -1): SLR < -2 (0.25× or smaller). Grey: no value (requirements for a reliably measured target not met). Calculated in Affymetrix comparison analysis (MAS5.0 algorithm) and at least one present call in the two síngle array analyses compared in the comparison analysis.

The alteration of gene expression related to apoptotic pathways correlated to the categorisation given above. Low responders also did also not respond with relevant gene expression changes of "apoptosis genes" whereas the high responders showed a significantly different gene expression profile concerning apoptosis related genes compared to the untreated control. In all, we found 464 genes with expression changes that are related to apoptotic pathways. The single genes that were differentially expressed in the medium and high responder group only partly overlapped with the low responder group. The heterogeneity of the response patterns of apoptotis related genes is illustrated in figure 3.

**Figure 4:**
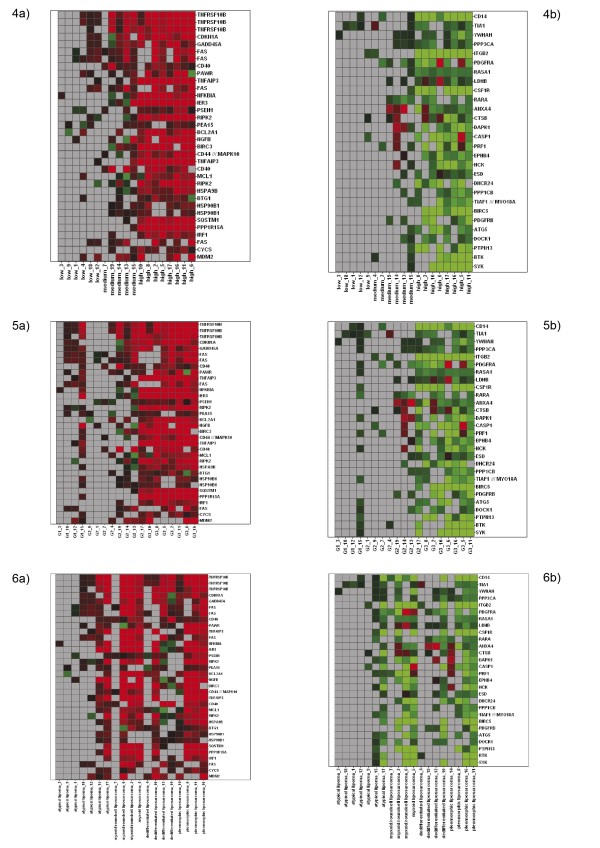
**Heatmaps of expression changes of selected genes associated with apoptotic pathways after incubation with doxorubicin for 24 h ordered by responder group (5 a,b), grading (6 a,b), and histologic subtype (7 a/b). **Expression changes as determined by comparison analysis were considered only if the probeset showed at least one P detection in untreated/treated sample pairs. Excluded expression changes are shown in grey. Only probesets with expression changes in 7 or more samples are given. Horizontal rows represent individual probesets. Vertical columns represent individual samples (left to right: low responders to high responders); gene symbols are indicated to the right. Gene expression changes are indicated by a continuous scale; the brightest red indicating ≥ 4-fold up-regulation; and, brightest green ≥ 4-fold down-regulated gene expression relative to the untreated control sample.

Although the diversity of changes in gene expression was large, some apoptosis related genes showed similar expression changes in the tumor samples, especially the high grade tumors (G2 and G3) or high responders. Figure 4 focus on expression changes in these genes. The apoptosis related genes most often affected by doxorubicin treatment are mentioned below. Due to their large number, we only refer to the genes that were differentially (up- or down-) regulated in more than 50% of the probes (samples). Some of the genes that were found up-regulated in the majority of the probes (tumors) could be found down-regulated in some other samples and vice versa. The heatmaps provided illustrate the similarity of the expression of these selected genes in correlation to responder group, grading, and histological subtype (fig. 4. The intrinsic and the extrinsic apoptotic pathway, as well as transcription factors and genes, so far only marginally associated with apoptotic pathways. A summary of the genes and their expression changes is given in table 2 and 3.

**Table 2:** Summary of the genes that were up-regulated by doxorubicin treatment, including the log ratios

Samples with increased expression (n)	Gene symbol	Mean log ratio	Range of log ratio	Samples with decreased expression (n)	Mean log ratio	(Range of) log ratio
15	TNFRSF10B	1.96	0.41/4.49	0	-	-
12	CDKN1A	1.85	0.59/4.10	1	-	-0.99
12	GADD45A	1.44	0.58/2.88	1	-	-0.59
12	FAS	1.36	0.27/3.87	1	-	-0.28
12	CD40	0.94	0.39/2.11	0	-	-
11	PAWR	1.16	0.33/2.51	1	-	-1.25
11	TNFAIP3	2.16	0.23/3.67	0	-	-
10	NFKBIA	2.04	0.43/3.34	2	-0.57	-0.45/-0.69
10	IER3	3.81	0.71/6.08	2	-0.60	-0.36/-0.84
10	PSEN1	0.84	0.23/1.61	2	-0.46	-0.46
10	RIPK2	2.02	0.42/3.65	2	-0.60	-0.46/-0.74
10	PEA15	0.61	0.23/0.96	1	-	-0.68
10	BCL2A1	2.18	1.03/5.08	1	-	-0.98
10	NGFB	3.06	0.40/5.80	1	-	-1.12
10	BIRC3	2.06	0.63/3.23	1	-	-0.93
10	CD44	1.57	0.26/3.09	1	-	-0.56
9	MCL1	1.46	0.67/2.08	3	-0.65	-0.26/-1.21
9	HSPA9	1.25	0.46/1.95	2	-0.50	-0.21/-0.79
9	BTG1	0.77	0.31/1.50	1	-	-1.06
9	HSP90B1	0.80	0.21/1.10	0	-	-
9	SQSTM1	2.03	0.27/3.23	0	-	-
9	PPP1R15A	3.16	0.65/4.40	0	-	-
9	IRF1	1.38	0.60/2.94	0	-	-
9	CYCS	0.82	0.34/1.37	0	-	-
9	MDM2	1.76	0.33/3.58	0	-	-

Negative log ratios stand for down-regulated genes.

**Table 3:** Summary of the genes that were down-regulated by doxorubicin treatment, including the log ratios

Samples with decreased expression (n)	Gene symbol	Mean log ratio	Range of log ratio	Samples with increased expression (n)	Mean log ratio	Range of log ratio
11	CD14	-2.99	-0.27/-5.38	0	-	-
10	TIA1	-0.68	-0.24/-1.05	1	-	0.77
10	YWHAH	-0.72	-0.27/-1.80	0	-	-
10	PPP3CA	-0.69	-0.34/-1.14	0	-	-
10	ITGB2	-2.87	-0.58/-5.11	0	-	-
9	PDGFRA	-1.32	-0.42/-3.30	2	2.62	1.21/4.03
9	RASA1	-0.93	-0.42/-1.17	0	-	-
8	LDHB	-0.60	-0.32/-1.25	2	1.11	0.64/1,58
8	CSF1R	-3.05	-0.63/-5.08	0	-	-
8	RARA	-0.95	-0.51/-1.23	0	-	-
7	ANXA4	-1.10	-0.64/-1.16	4	1.37	0.84/2.33
7	CTSB	-0.93	-0.23/-1.97	3	0.94	0.82/1.13
7	DAPK1	-1.35	-0.59/-2.07	2	1,40	1.19/1.60
7	CASP1	-1.69	-0.71/-2.90	2	1.44	0.97/1.90
7	PRF1	-1.68	-0.54/-3.68	2	0.79	0.67/0.91
7	EPHB4	-1.30	-0.68/-2.34	1	-	0.30
7	HCK	-1.79	-0.39/-3.65	1	-	1.08
7	ESD	-0.76	-0.44/-0.95	1	-	0.36
7	DHCR24	-1.97	-0.75/-4.34	0	-	-
7	PPP1CB	-0.99	-0.62/-1.56	0	-	-
7	MYO18A/TIAF1	-1.23	-0.67/-2.02	0	-	-
7	BIRC5	-2.95	-1.17/-4.91	0	-	-
7	PDGFRB	-1.28	-0.61/-2.26	0	-	-
7	ATG5	-1.39	-0.61/-3.19	0	-	-
7	DOCK1	-0.86	-0.40/-1.23	0	-	-
7	PTPN13	-1.97	-0.69/-3.17	0	-	-
7	BTK	-1.78	-0.39/-3.28	0	-	-
7	SYK	-4.26	-0.34/-8.06	0	-	-

Positive log ratios stand for up-regulated genes.

The results of the microarray analysis were validated using rtPCR. The general Pearson correlation of the expression changes measured in all selected genes in the tumor probes was high (0.913). The correlation coefficients for the single candidate genes are given in table 4.

**Table 4:** Pearson coefficient calculated for the candidate genes describing the correlation of the gene expression changes measured microarray and rtPCR.

Gene symbol	Pearson coefficient
CD40	0.945
CDKN1A	1.000
FAS	0.813
GADD45A	0.848
ITGB2	0.999
NFKBIA	0.882
PAWR	0.913
PPP3CA	0.638
TNFAIP3	0.997
TNFRSF10B	0.998
YWHAH	0.370

## Discussion

Gene expression profiling has already been helpful in categorizing distinct subtypes of sarcomas by profile clustering [33-35] and identifying subtype specific changes in gene expression in liposarcoma, e.g. abnormal expression of cell cycle regulators in *FUS-DDIT3 *carrying liposarcomas [34,36-43] and even provided potential targets for new therapeutic agents like important mediators in cell cycle regulation, e.g. *MDM2 *[44-48].

Gene expression profiling studies on liposarcomas have already shown that this entity presents a somewhat similar expression pattern with malignant fibrous histiocytoma and leimyosarcoma [31,49] and that highly differentiated lesions cluster with lipoma whereas the dedifferentiated tumors cluster with myxoid/round cell liposarcomas [50]; however no clear correlation between expression patterns and histological subtype could be detected [37]. Another difficulty in the assessment of gene expression profiles is the inter- and intra-tumoral heterogeneity. Several subtypes with different expression patterns and histologic features can often be found within one same tumor [51,52].

Liposarcomas are classified into several types based on histological findings and cytological aberrations – well differentiated (atypical lipoma), dedifferentiated, myxoid, round cell tumors and pleomorphic. The risk of distant metastasis grows with the grading of the lesion to up to 75% in pleomorphic sarcoma. Myxoid tumors with a greater than 5% round cell component, most dedifferentiated, and pleomorphic liposarcomas are considered high grade lesions [4,53-55].

There are no markers to clearly identify liposarcoma cells. S100, CD34, and others may be helpful (as well as cytogenetic techniques) as they can identify aberrations indicating myxoid/round cell sarcoma; however, they cannot identify liposarcoma cells with absolute certainty [56]. In our series, we relied on a proper tumor dissection and preparation of the specimens to ensure, the isolated tissue mainly consisted of liposarcoma cells as previously described by Sreekantaiah et al. [57] and Lehnhardt et al [58], but have to admit that results may be partly falsified by residual tumor stroma cells accidentally co-cultivated within the liposarcoma samples.

Liposarcoma cells showed diverse gene expression patterns before and after incubation with doxorubicin. Tumors of the same histologic subgroup did not cluster together concerning their overall gene expression. The correlation of the number of differentially regulated genes to the tumor grading, and, to a lesser extent, to the histological subgroup after doxorubicin incubation, may be caused by the tumor associated up-regulation of cell metabolism and the therefore greater effect of any interference. The overall expression patterns and the ones of the apoptosis related genes were also very heterogenous (fig. 2, 3). This finding is concordant to the results of other studies with soft tissue sarcoma cells [59] and may partly be explained by the known inter- and intratumoral heterogeneity in soft tissue sarcomas [51,60].

Interestingly the myxoid and the myxoid/round cell liposarcomas clustered together in figure 2 and 3 except from the tumor 7 that was pre-treated with chemotherapy. If that is a coincidence or may be interpreted as a kind of selectional process that could have eliminated the high grade parts of the tumor leaving the residual to cluster closer to the low grade sarcomas can not be determined.

The predescribed low clinical response rates of low grade sarcoma correlate to our findings that low grade liposarcoma, especially atypical lipoma, showed almost no response to doxorubicin on gene expression level [61]. However, some expression changes in response to doxorubicin treatment, observed especially in the high responder and in the high grade group were similar and are focussed on the figure 4. According to the large number of apoptosis related genes, we identified the first five ones that were differentially expressed in more than 50% of the samples and limit further explanation to these with special reference to apoptotic function in sarcoma or previous reports concerning doxorubicin treatment, although the understanding of their exact functions is limited. The up-regulated genes beyond the mentioned five are summarized in table 5.

**Table 5:** Summary of genes beyond the ones already mentioned in the text that were found to be up-regulated by doxorubicin treatment in more than 50% of the probes.

Gene symbol	Gene name	Probes upregulated	Additional information	Apoptotic function
PAWR	PRKC (protein kinase C) apoptosis WT1 (Wilms tumor gene) regulator protein	11	STS with high WT1 mRNA expression levels have poorer outcome than those with low levels [89,90]. Ecteinascidin that has been shown to be effective against STS also increases expression of PAWR [92]. PAWR inhibits the PKC (atypical protein kinase)-NF-(kappa)B (nuclear factor-(kappa)B)-XIAP pathway [91].	proapoptotic
TNFAIP3	tumor necrosis factor, alpha-induced protein 3	11	TNFAIP3 down-regulates the TNF-α-induced NFκB signalling pathway [93,94].	antiapoptotic
NFKBIA	nuclear factor of kappa light polypeptide gene enhancer in B-cells inhibitor alpha	10	The doxorubicin analogon DA-125 reduces proliferation in HT1080 fibrosarcoma cells through a NFKB dependent pathway [96,97]. MDM2 (upregulated in 9 samples in our series) is overexpressed in several liposarcoma subtypes [58] and increases NFKB activity in a p53 dependent manner and thereby leads to doxorubicin resistance [98].	proapoptotic/antiapoptotic
IER3	immediate early response 3	10	IER3 function is increased by p53, that is induced by doxorubicin. IER3 is involved in cell cycle arrest and programmed cell death [99,100].	proapoptotic
PSEN1	presenilin 1	10	Effects are mediated via Bcl-2 interaction [101].	proapoptotic
RIPK2	receptor-interacting serine-threonine kinase 2	10	RIPK2 is cell death inducing and NFKB activating, via caspase 1 activation [102,103].	proapoptotic
PEA15	phosphoprotein enriched in astrocytes 15	10	RPEA15 is regulating caspase-3 function in epidermal cells [104], but has not yet been associated with doxorubicin treatment or apoptosis in sarcoma cells.	antiapoptotic
BCL2A1	BCL2 (B-cell CLL/lymphoma 2)-related protein A1	10	BCL2A1 stabilizes the mitochondrial membrane [105,106].	antiapoptotic
NGFB	nerve growth factor, beta polypeptide	10	NGF reduces apoptosis induced by chemotherapeutics in sarcoma cells [109].	antiapoptotic
BIRC3	baculoviral IAP repeat-containing 3	10	BIRC3 is associated with chemotherapy resistance in Ewing sarcoma, rhabdomyosarcoma [110] and prostatic cancer [111].	antiapoptotic
CD44	cell surface glycoprotein CD44	10	CD44 is a proapoptotic factor in FAS mediated apoptosis in sarcoma cells [112], but is also connected to cancer drug resistance [113]. CD44 has successfully been used as a target for liposomal encapsuled doxorubicin [114].	proapoptotic/antiapoptotic

### Up-regulated genes

As previously shown, doxorubicin could overcome TRAIL resistance in a variety of sarcoma cell lines. An up-regulation of the *TRAIL-R2*, increasing the susceptibility to apoptosis inducing agents such as TRAIL, may be a possible explanation [62-65], as we found the Trail receptor 2 gene expression increased in 15 probes.

The second most frequently up-regulated gene was cyclin-dependent kinase inhibiton 1A (*CDKN1A*), a downstream target of *p53*, has has already been shown to be involved in cell cycle arrest and apoptosis induction by doxorubicin in sarcoma cells. *CDKN1A *can act as apositive regulator of senescence-like terminal proliferation arrest, but its function seems neither sufficient nor absolutely required for a treatment response to doxorubicin in tumor cells, especially soft tissue sarcoma [66-70].

*GADD45A*, a potent inhibitor of the c-Jun N-terminal kinase (JNK) cascade and *NFKBIA*, inhibits transcription factors associated with tumor growth [71-74] and was up-regulated by doxorubicin in 12 probes. In a variety of soft tissue sarcoma cell lines GADD45A was found to increase cell cycle arrest and apoptosis {Zhu, 2008 #165}. For rhabdomyosarcoma, increased *GADD45A *has previously been associated with less aggressive tumor behaviour [75].

*FAS*, a member of the tumor necrosis factor receptor family, seems to be an important mediator in doxorubicin induced apoptosis. Its effects were shown to be dependant on metalloproteinases in soft tissue sarcoma and Ewing sarcoma. These metalloproteinases have been associated with aggressive tumor behaviour and may promote invasiveness and the occurrence metastasis of malignant cells [76-78].

Another member of the TNF receptor superfamily, *CD40*, was also up-regulated by doxubicin in our experiments. To the authors' knowledge, *CD40 *has not yet been associated with doxorubicin treatment in liposarcoma, but its expression in soft tissue sarcoma was associated with an unfavourable outcome [79], whereas Lodge et al. could show a beneficial effect of antibody mediated CD40 activation in elimination of fibrosarcoma in nude mice [80].

### Down-regulated genes

Among the down-regulated genes related to apoptotic pathways *CD14*, a receptor marking apoptotic cells was found in 8 probes. Increased *CD14 *has been associated with apoptosis induction and cell clearance, especially mediated by macrophages, but is expressed by a variety of other cells too [81-83]. Its role in terms of cell death mediation in sarcoma cells has not yet been examined, therefore interpretation of this finding remains difficult.

*TIA-1*, which encodes an RNA-binding protein with translation-regulatory functions has already been reported to be up-regulated in tumor specimens post-treatment with TNF alpha in soft tissue sarcomas. It was further hypothesized that *TIA-1 *could mediate death receptor mediated apoptosis in soft tissue sarcoma and that its overexpression might sensitize endothelial cells to proapoptotic stimuli present in the tumor microenvironment and enhance NK cell cytotoxic activity against cancer cells [84].

*YWHAH*, or 14-3-3 eta, is a member of the dimeric 14-3-3 family of signal transduction proteins that specifically binds to phosphorylated serine on a variety of signalling molecules, such as *Bcl-2*, *MDMX*, and *Bax*, thereby promoting cell survival and acting antiapoptotic in several tumor cells [85-87]. On the other hand, it is supposed to be associated with tumorigenesis through its binding interaction with gremlin1 [88]. Therefore the issue of further studies should be awaited before interpreting this finding.

*PPP3CA *(*CCN1*/*Cyr61*) is susceptible to various growth factors and promotes cell proliferation, adhesion, and differentiation and plays important roles in angiogenesis. Additionally, *PPP3CA *has been associated with tumorigenesis. It was reported that *PPP3CA *exerts its functions via interacting with integrins as well as heparan sulfate proteoglycan. By activating NF-kappaB and tyrosine kinase signalling pathways, *PPP3CA *is not only able to control cell growth, but also induce or suppress apoptosis in a cell type-specific manner [89,90]. To the authors' knowledge, it has so far not been reported in context with liposarcoma or doxorubicin treatment.

Integrin B2 (*ITGB2*) is known to play a role in mediating apoptosis [91] and chemotherapy resistance. Although it is widely attributed to white blood cells, it is also expressed in a variety of other benign and malignant cells and seems to play a major role in cell invasion and migration [92].

## Conclusion

In summary, pro- and antiapoptotic genes were found up- as well as down-regulated with a dominance of up-regulation of proapoptotic genes. The heterogeneous expression profiles reflect the heterogeneous reaction of liposarcomas to doxorubicin therapy. A lot of genes we found differentially expressed have not yet been associated with apoptosis in liposarcoma or doxorubicin treatment. Therefore it is not possible to relate our findings to other studies. Our study shows that the low clinical response rates of highly differentiated liposarcoma correlate to minimal changes in the expression patterns and that only high grade tumors, especially myxoid/roundcell and pleomorphic tumors, respond to doxorubicin on gene expression level. In most cases, this response seems to be based on an increase of the extrinsic pathway such as TRAIL Receptor 2 and FAS but also members of the intrinsic pathway such as BCL2A1 were found to be differentially expressed. Interestingly several factors (NFKBIA, GADD45A, RIPK2, and PAWR) point to the *NFKB *transcriptional factor as possible mediator of doxorubicin effects.

Our results indicate that gene expression profiling may be a promising approach to improve the understanding of the diverse modes of programmed cell death in liposarcoma following doxorubicin treatment and can provide a molecular basis for new chemotherapeutic strategies.

## Competing interests

The authors declare that they have no competing interests.

## Authors' contributions

AD developed the study design, coordinated the work, interpreted the data and prepared the manuscript. LKH carried out and interpreted the microarrays. AMC carried out statistical analyses, have given substantial contribution to conception and design as well manuscript preparation. OM improved the study design and corrected the manuscript. HJ prepared the figures and gathered patients data. HHH was helpful in preparing the manuscript and conceived the work. HUS was helpful in preparing the manuscript and conceived the work. ML carried out cell culture and developed the idea, study design and conceived the work

## Pre-publication history

The pre-publication history for this paper can be accessed here:


